# Chemically Induced Fluorescence Switching of Carbon-Dots and Its Multiple Logic Gate Implementation

**DOI:** 10.1038/srep10012

**Published:** 2015-05-06

**Authors:** Namasivayam Dhenadhayalan, King-Chuen Lin

**Affiliations:** 1 Department of Chemistry, National Taiwan University, and Institute of Atomic and Molecular Sciences, Academia Sinica, Taipei 106, Taiwan

## Abstract

Investigations were carried out on the carbon-dots (C-dots) based fluorescent off - on (Fe^3 + ^- S_2_O_3_^2−^) and on - off (Zn^2 + ^- PO_4_^3−^) sensors for the detection of metal ions and anions. The sensor system exhibits excellent selectivity and sensitivity towards the detection of biologically important Fe^3 + ^, Zn^2 + ^ metal ions and S_2_O_3_^2−^, PO_4_^3−^ anions. It was found that the functional group on the C-dots surface plays crucial role in metal ions and anions detection. Inspired by the sensing results, we demonstrate C-dots based molecular logic gates operation using metal ions and anions as the chemical input. Herein, YES, NOT, OR, XOR and IMPLICATION (IMP) logic gates were constructed based on the selection of metal ions and anions as inputs. This carbon-dots sensor can be utilized as various logic gates at the molecular level and it will show better applicability for the next generation of molecular logic gates. Their promising properties of C-dots may open up a new paradigm for establishing the chemical logic gates via fluorescent chemosensors.

Recently, chemical based molecular logic gates received extensive attention due to its fascinating significance for the development of molecular-scale electronic devices, which perform Boolean logic operations in response to chemical inputs. So far several groups have demonstrated the construction of the molecular logic gates by using different materials such as nucleic acids^1^,^2^,^3^,^4^,^5^,^6^, proteins^7^,^8^, and organic molecules^9^,^10^,^11^,^12^. Recently, Feng *et al.* reported that spermine-functionalized C-dots can influence DNA structure to induce DNA B-Z transition and perform DNA logic operations^13^. Despite the promising performance of the DNA molecule, it is necessary to investigate the new materials which show better applicability for the next generation molecular logic gates. Herein we report the new C-dots based logic gates and illustrate their application for the logic functions of OR, XOR and IMP. C-dots exhibits tremendous characteristics such as non-toxicity, biocompatibility, photostability, facile, rapid and low cost for synthesis, easy for surface modification, and excellent luminescence properties^14^,^15^,^16^,^17^,^18^. Due to these unique properties, developing C-dots as a new material to perform logic operations should be worthwhile.

Among several methods (e.g. luminescence, colorimetric and electrochemical), photoluminescence is a broadly used characterization technique for logic devices because of its characteristics such as high sensitivity, simple and fast response^5^,^10^,^19^,^20^,^21^. We establish the C-dots based fluorescence off-on and on-off sensors which sense the metal ions as well as anions in pH 7.2 HEPES-buffered water solution. In this study, carboxylate and amine functionalized C-dots (for convenience, C-dots/COOH and C-dots/NH_2_ named as C-dots1 and C-dots2, respectively, thereafter) are acted as fluorescent probes and several metal ions (Ti^2 + ^, Cr^2 + ^, Mn^2 + ^, Fe^2 + ^, Fe^3 + ^, CO^2 + ^, Ni^2 + ^, Cu^2 + ^, Zn^2 + ^, Sn^2 + ^, Cd^2 + ^, Hg^2 + ^ and Pb^2 + ^) and anions (F^−^, Cl^−^, Br^−^, I^−^, CH_3_COO^−^, SO_3_^2−^, SO_4_^2−^, S_2_O_3_^2−^, NO_2_^−^, NO_3_^−^, PO_4_^3−^ and HPO_4_^2−^) are tested. The obtained results reveal that the C-dots1 shows selectivity towards Fe^3 + ^ and S_2_O_3_^2−^ whereas C-dots2 shows selectivity towards Zn^2 + ^ and PO_4_^3−^ ions. Fe^3 + ^ and Zn^2 + ^ are involved in major functions of complex chemistry and biology of the human brain and also play vital role in various biological systems such as in cellular metabolism, gene expression and neurotransmission^22^,^23^,^24^,^25^. Similarly, the anions also play predominant roles in biological, chemical and environmental processes^26^. Thiosulphate have many important physiological features and are used in medicine and industry applications^27^,^28^,^29^, and the phosphate anions are one of most important constituents of living systems and their derivatives play pivotal roles in biological systems^30^,^31^. Thus, there is an essential task to the developing a facile detection method for metal ions and anions in biological media to better understand its physiological roles in chemistry and biological studies. Based on the sensing results we implemented the molecular logic gates behavior by observing the fluorescence response, i.e., change in the fluorescence intensity with varying the chemical inputs such as metal ions and anions. In comprehensive, the most important observation is the construction of different logic functions which mainly depend on the functional group present on the C-dots surface, interaction between the C-dots and metal ions/anions, and the reaction between the metal ions and anions.

## Results and Discussion

**Figure 1:**
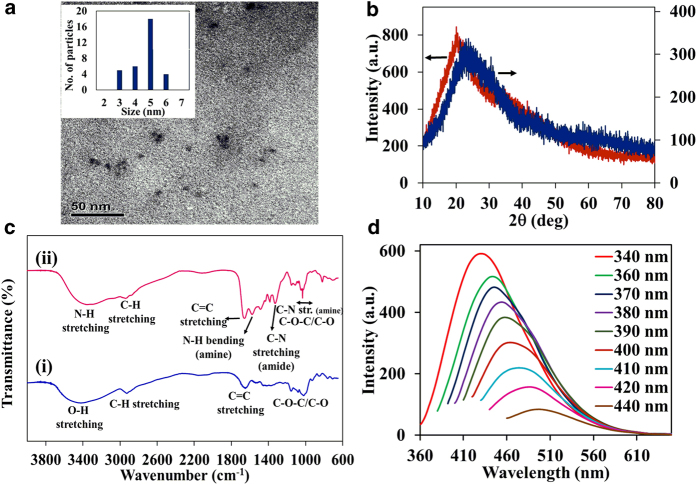
(**a**) TEM image of C-dots2, inset shows size distributions; (**b**) XRD patterns of the C-dots1 (red line) and C-dots2 (blue line); and (**c**) FTIR spectra of C-dots1 (i) and C-dots2 (ii); (**d**) Fluorescence spectra of C-dots2 with different excitation wavelengths.

The C-dots1 and C-dots2 were prepared through carbonization of rice as a carbon precursor by a one-step microwave-assisted method. The estimated quantum yield of C-dots1 and C-dots2 are 3 and 11% respectively obtained by using quinine sulphate as the reference. The size and morphology of C-dots were observed by transmission electron microscopy (TEM). The TEM image clearly indicates that the formed C-dots were spherical in shape with average diameters of 5 nm and well dispersed to each other (Fig. 1a). The XRD pattern (Fig. 1b) showed a broad diffraction peak appeared at ~20 ° (d = 0.45 nm) and ~24 ° (d = 0.38 nm) for C-dots1 and C-dots2 respectively. The calculated interlayer distance is lower than that of graphite (d = 0.34 nm). The diffraction peak of C-dots1 shifted to a lower degree may be attributed to the reduction of lattice parameters of a unit cell of the C-dots. Further, the FTIR spectrum was recorded to determine the surface functional groups of C-dots (Fig. 1c). Both C-dots exhibit a broad peak with the maxima at ~3400 cm^−1^ corresponding to the O-H and N-H stretching vibration of carboxylic acid and amine groups respectively. In addition, both C-dots display C-H vibration at ~2930 cm^−1^, C = C vibration at ~1650 cm^−1^ and C-O-C vibration at ~1100 cm^−1^. In C-dots2, three strong absorption peaks observed at 1575, 1385 and 1111 cm^−1^ indicate the presence of N-H bending vibration (amine), amide C-N and amine C-N stretching bands, thereby revealing that ethylenediamine (EDA) in reaction with carboxylic groups on the surface results in the formation of amide group^32^. FTIR results confirm that the C-dots have various surface functional groups such as hydroxyl, carbonyl, epoxy, carboxylic and amine groups. The elemental analysis reveals that the C-dots1 is composed of C 40.25, H 6.50, N 1.66 and O 51.54%, whereas the C-dots2 is composed of C 38.40, H 6.80, N 11.34 and O 41.81%. A decrease in an oxygen content of C-dots2 further confirms the reaction occurs between -COOH and EDA on the surface of C-dots2.

The absorption spectrum of C-dots1 shows strong absorption band peaking at ~280 nm with a tail extended to 500 nm. Besides, the C-dots2 exhibits strong absorbance in the region of above 300 nm compared with that of C-dots1. In both C-dots, the observed band at ~280 nm is attributed to the π–π* transition corresponding to carbon core C = C units whereas the shoulder at ~350 nm is due to the n–π* transition corresponding to the carbonyl/amine functional groups on the surface^14^,^32^. Fluorescence spectra of C-dots were recorded at different excitation wavelength and the resultant fluorescence spectra obviously represent that C-dots have multi-emission nature and depending on the excitation wavelength (Fig. 1d). Moreover, the fluorescence maximum and intensity was found to be dependent on the excitation wavelengths. Upon C-dots excitation varying from 320 to 450 nm, the emitted fluorescence maximum was red shifted from ~420 to 520 nm and higher fluorescence intensity was observed at 340 nm excitation.

**Figure 2:**
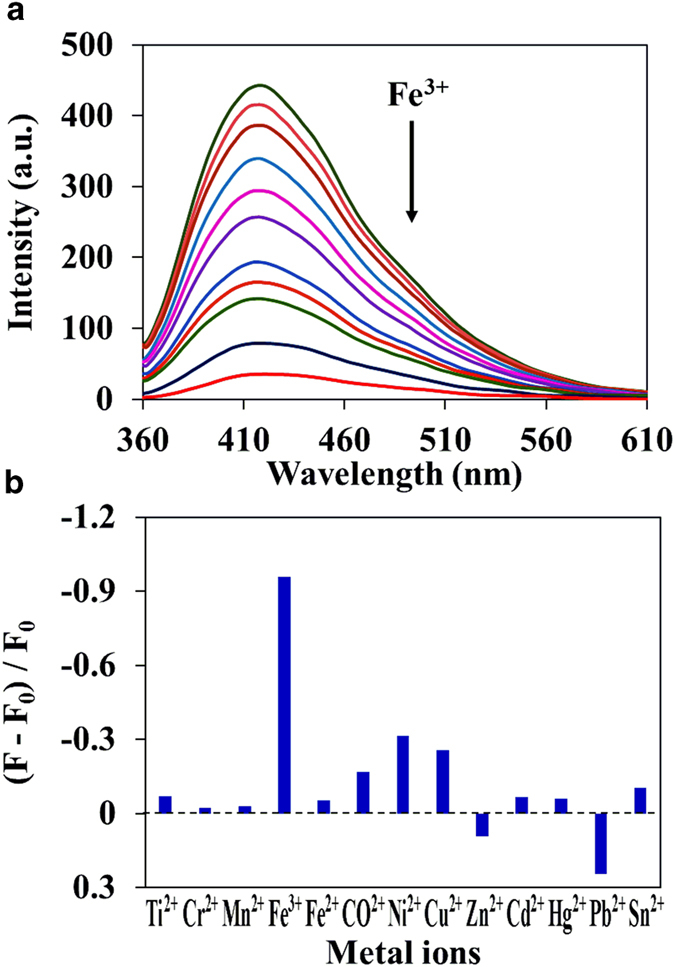
**a**) Fluorescence spectra of C-dots1 (0.002 mg mL^−1^) with different concentration of Fe^3 + ^ (0 to 2.5 × 10^−4^ M) in pH 7.2 HEPES-buffered water solution. (**b**) Fluorescence response of C-dots1 in the presence of different metal ions. λ_ex_ = 340 nm.

Next, we determined the influence of metal ions on the C-dots fluorescence intensity by using fluorescence titration method. The fluorescence titration of C-dots with different concentration of metal ions in pH 7.2 HEPES-buffered water was used throughout all the experiments. Despite the same experimental conditions, the two different functionalized C-dots exhibits different sensitivity and selectivity for metal ions. For C-dots1, Fe^3+^ ions show significant effect on fluorescence quenching among the metal ions studied. The fluorescence intensity was found to decrease with increasing concentration of Fe^3+^ ions, but peaking at the same position even in the presence of highest concentration of Fe^3+^ ions (Fig. 2). The observed fluorescence quenching of C-dots1 may be due to non-radiative electron transfer from the excited state of the C-dots to the d-orbital of Fe^3+^ ion^18^,^33^. The fluorescence quenching process was analyzed by using Stern-Volmer (S-V) relation (F_0_/F = 1 + K_SV_ [Q] = 1 + k_q_ τ_0_ [Q])^34^. The S-V plot shows linearity in the concentration range of 0 – 100 μM, yielding K_SV_ of 2.5 × 10^4^ M^−1^. The quenching rate constant k_q_ was calculated to be 5.15 × 10^12^ M^−1^ s^−1^, which reveals that the high efficiency of quenching process in the excited state. The determined k_q_ value is much higher than the diffusion-controlled quenching limit, suggesting that the Fe^3+^ ions plausibly coordinate with -COOH groups on the surface of C-dots1^18^,^33^. When the concentration keeps increasing, the S-V plot begins to deviate from linearity, indicating that the observed quenching process may be due to both dynamic and static process.

**Figure 3:**
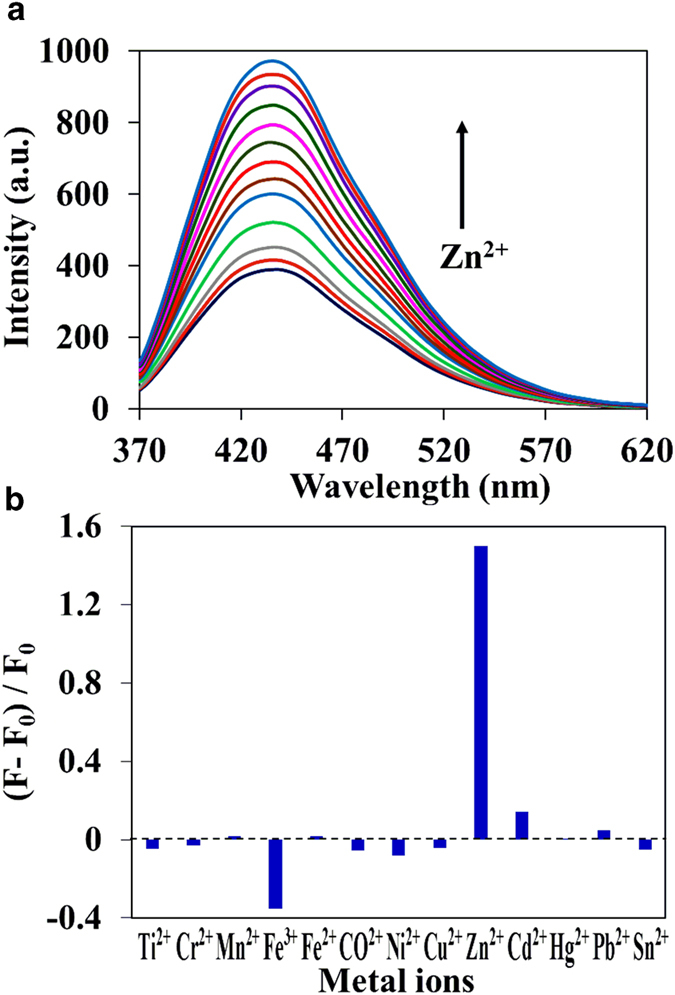
**a**) Fluorescence spectra of C-dots2 (0.002 mg mL^−1^) with different concentration of Zn^2 + ^(0 to 2.5 × 10^−4^ M) in pH 7.2 HEPES-buffered water solution. (**b**) Fluorescence response of C-dots2 in the presence of different metal ions. λ_ex_ = 340 nm.

In the case of C-dots2, drastic enhancement of fluorescence intensity was observed in the presence of Zn^2+^ ions (Fig. 3). The fluorescence intensity increased with increasing the concentration of Zn^2 + ^, but the peak position did not change. The observed fluorescence enhancement is probable due to the higher affinity of Zn^2+^ with the nitrogen atoms of amine groups on the C-dots surface. Upon addition of other metal ions including Fe^2+^, Cr^2+^, Ni^2+^, CO^2+^, Cu^2+^, Hg^2+^, Pb^2+^, Ti^2+^, Mn^2+^, Cd^2+^ and Sn^2+^, the fluorescence intensity and maximum of both C-dots do not altered much even in the presence of highest concentration of metal ions (2.5 × 10^−4^ M).

**Figure 4:**
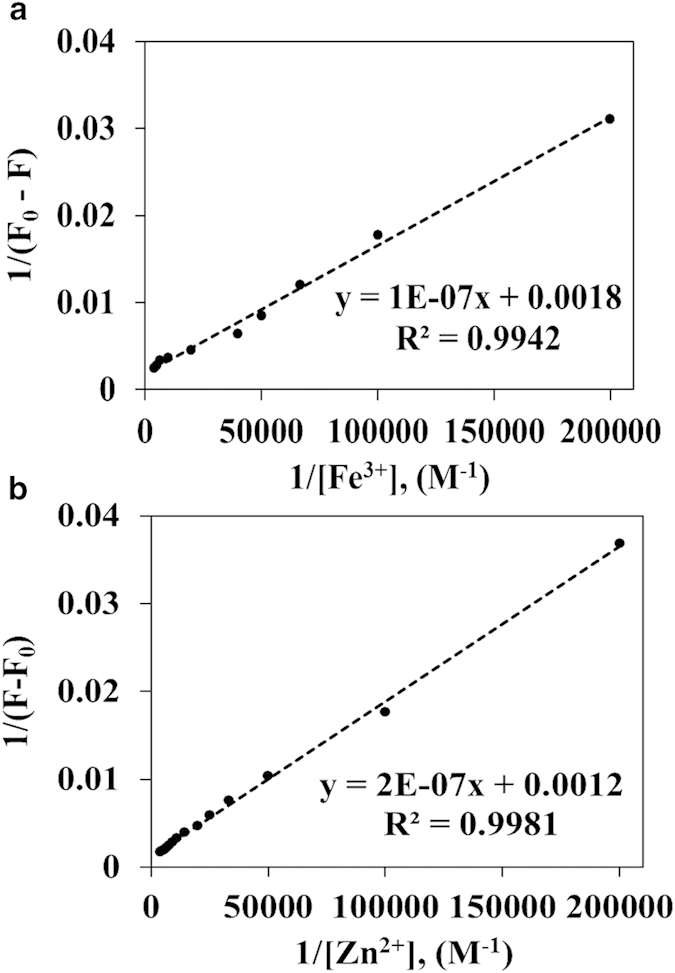
Benesi-Hildebrand plot of 1/F−F_0_ versus 1/[M^n + ^] for the (**a**) C-dots1/Fe^3 + ^and (**b**) C-dots2/Zn^2 + ^systems.

Further, the stoichiometry for interaction of C-dots with metal ions and the association constant was determined by using the Benesi-Hildebrand method based on the changes in the fluorescence intensity^35^. The Benesi-Hildebrand plot of 1/(F − F_0_) versus 1/[M^n+^] exhibits a straight line for both C-dots, which confirms the 1:1 stoichiometry of C-dots with metal ions (Fig. 4). The association constant (log K_a_) was determined by dividing the intercept by the slope of the straight line, yielding value of 4.05 and 3.78 for C-dots1/Fe^3+^ and C-dots2/Zn^2+^ systems respectively. The detection limit of the C-dots was determined on the relation 3(σ/slope), where σ is the standard deviation of the response. A good linear correlation was observed over the concentration range of 0 – 50 μM. The measured detection limits are 0.78 × 10^−7^ and 1.63 × 10^−7^ M for C-dots1/Fe^3 + ^ and C-dots2/Zn^2 + ^ systems, respectively. From the obtained results, the C-dots sensors appear to be highly selective and sensitive for the detection of metal ions. In particular, C-dots1 shows more affinity towards Fe^3+^ detection which is similar to previously reported literatures^18^,^33^,^36^,^37^,^38^. Recently, Zhang *et al.* reported that the quinolone derivative-functionalized carbon dots exhibited good response towards the sensing of Zn^2+^ ion^39^. However, to the best of our knowledge, this is the first report of using C-dots for label-free detection of Zn^2+^ with high selectivity.

**Figure 5:**
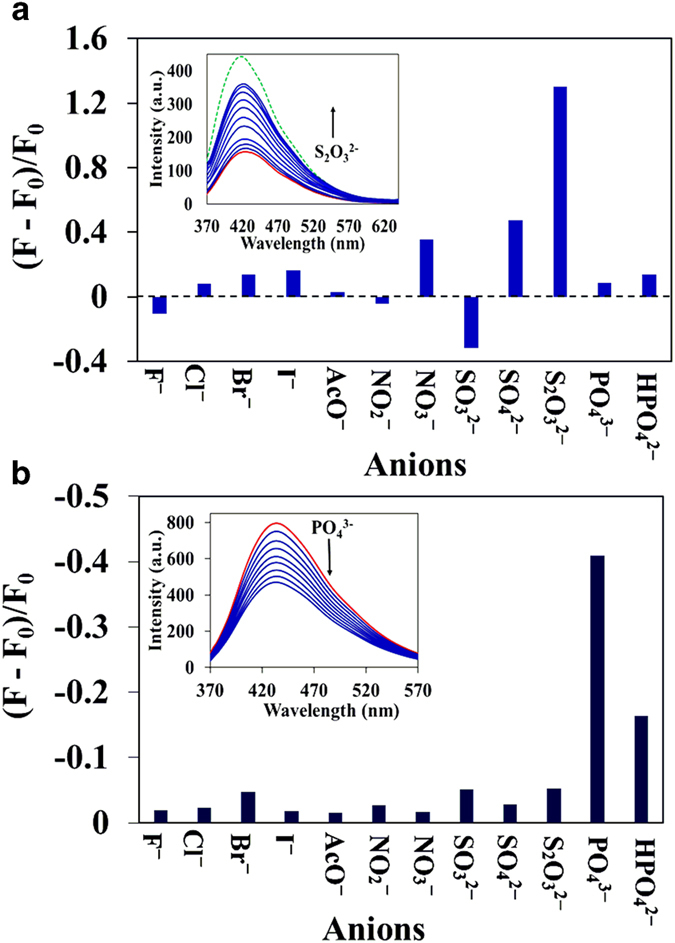
**a**) Fluorescence response of C-dots1/Fe^3 + ^in the presence of different anions; Inset: Fluorescence spectra of C-dots1/Fe^3 + ^in the absence (red line) and presence (blue lines) of different concentration of S_2_O_3_^2−^ (0 to 1.25 × 10^−3^ M); Green dotted line: fluorescence spectrum of C-dots1 alone. (**b**) Fluorescence response of C-dots2/Zn^2 + ^in the presence of different anions; Inset: Fluorescence spectra of C-dots2/Zn^2 + ^in the absence (red line) and presence (blue lines) of different concentration of PO_4_^3−^ (0 to 1.25 × 10^−3^ M). λ_ex_ = 340 nm.

In addition to the sensing of metal ions, we examined the sensing of anions for both C-dots/M^n + ^ systems based on the fluorescence on-off and off-on method. To investigate the selectivity of anion detection, the fluorescence spectra of C-dots/M^n + ^ systems in the presence of biological relevant anions including F^−^, Cl^−^, Br^−^, I^−^, CH_3_COO^−^, SO_3_^2−^, SO_4_^2−^, S_2_O_3_^2−^, NO_2_^−^, NO_3_^−^, PO_4_^3−^ and HPO_4_^2−^ were recorded (Fig. 5). Interestingly, upon addition of anions the quenched fluorescence was recovered for C-dots1/Fe^3+^ system whereas the enhanced fluorescence was quenched for C-dots2/Zn^2+^. It was found that the quenched fluorescence of C-dots1/Fe^3+^ gradually recovers with increasing concentration of S_2_O_3_^2−^ ion (Fig. 5a). Approximately 80% of the fluorescence at 420 nm was recovered by the addition of 1.25 M S_2_O_3_^2−^, and this recovery percentage can be increased with further increase of the concentration. The detection limit of the C-dots1/Fe^3+^ for S_2_O_3_^2−^ was calculated to be about 8.47 × 10^−6^ M. Similarly, to study the selectivity and sensitivity of C-dots2/Zn^2+^ for anion detection, the fluorescence spectra of C-dots2/Zn^2+^ were recorded in the presence of various anions and the plot of selectivity is shown in Fig. 5b. The results demonstrate that this sensor system displays good selectivity towards PO_4_^3−^ anions. The fluorescence intensity of the ensemble solution was quenched on the addition of PO_4_^3−^. The detection limit of C-dots2/Zn^2+^ system for the sensing of PO_4_^3−^ was calculated to be 6.89 × 10^−6^ M. In contrast, other anions show very minor influence on the fluorescence intensity of the C-dots2/Zn^2+^ system except for HPO_4_^2−^ which shows moderate response.

**Figure 6:**
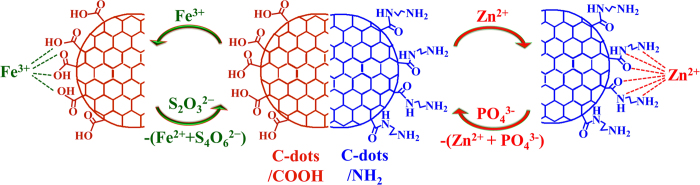
Schematic representation for the sensing process of metal ions and anions with C-dots.

The detection process of metal ions and anions is shown in Fig. 6. The C-dots1 contains ample amount of carboxylate groups on the surface and Fe^3+^ ions shows a certain affinity to the oxygen atoms which resulted in the association between the Fe^3+ ^ ions and carboxylate groups. On the other hand, owing to strong interaction between Fe^3 + ^ and S_2_O_3_^2−^ ions, the associated Fe^3 + ^ ions react with S_2_O_3_^2−^ to forms free C-dots1. Due to above two facts, the C-dots1 system exhibited off-on fluorescence behavior. Similarly, Zn^2 + ^ displays an affinity with nitrogen and oxygen atoms; as a consequence the C-dots2 fluorescence was enhanced due to association between Zn^2 + ^ and amine groups present on the surface. Then, the associated C-dots2/Zn^2 + ^ moieties could be disassociated by the addition of PO_4_^3−^ due to affinity between Zn^2 + ^ and oxygen atoms of PO_4_^3−^, which leads to turn-off fluorescence of C-dots2. These results proved that this off-on and on-off fluorescence process depend on the functional groups present on the C-dots surface and affinity nature of metal ion with anions.

Based on the observations in the sensing studies of metal ions and anions by C-dots, we demonstrate individual logic gates such as YES, NOT, IMP, OR and XOR. The performance of various logic gate functions depend on the association and dissociation of metal ions (Fe^3 + ^or Zn^2 + ^) and anions (S_2_O_3_^2−^ or PO_4_^3−^) with C-dots and their corresponding fluorescence intensity emitted by C-dots. Experiments were performed on C-dots with the four possible input combinations (0,0; 1,0; 0,1; 1,1) for multiple logic gate operations. In logic gates implementation, metal ion (Fe^3 + ^or Zn^2 + ^) and anion (S_2_O_3_^2−^ or PO_4_^3−^) are used as input 1 and input 2 respectively, and the fluorescence signal as output. The absence and association of these ionic inputs with C-dots defines as 0 and 1 states respectively. The output defined as 1 and 0 corresponds to strong and weak fluorescence response^9^. The simplest logic gates NOT and YES were achieved by giving metal ions (Fe^3 + ^or Zn^2 + ^) as input 1 to the C-dots, in the absence of input 2 (anions), and producing a low or high fluorescence output signal. The fluorescence of C-dots1 quenched in the presence of Fe^3 + ^ indicates that the output is 0 (low), on the contrary, fluorescence of C-dots2 enhanced in the presence of Zn^2 + ^ indicates that the output is 1 (high). These results correlate well with the function of NOT and YES logic gates respectively^9^.

**Figure 7:**
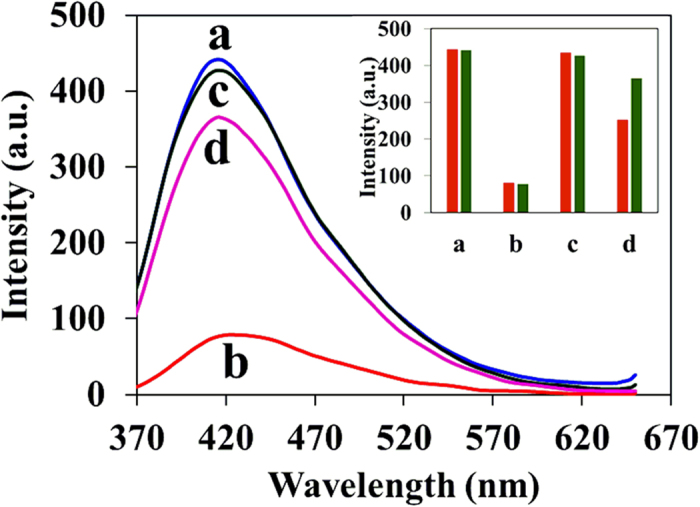
Fluorescence response of C-dots1 in the absence and presence of Fe^3 + ^(2.5 × 10^−4^ M) and S_2_O_3_^2−^ (3.0 × 10^−3^ M); **a**: C-dots1, **b**: C-dots1/Fe^3 + ^, **c**: C-dots1/S_2_O_3_^2−^, **d**: C-dots1/Fe^3 + ^/S_2_O_3_^2−^. Inset: Bar diagram showing the fluorescence intensity changes for different combination of inputs, Red color: in the presence of low concentration of S_2_O_3_^2−^ (2 × 10^−4^ M), Green color: in the presence of high concentration of S_2_O_3_^2−^ (3.0 × 10^−3^ M).

**Figure 8:**
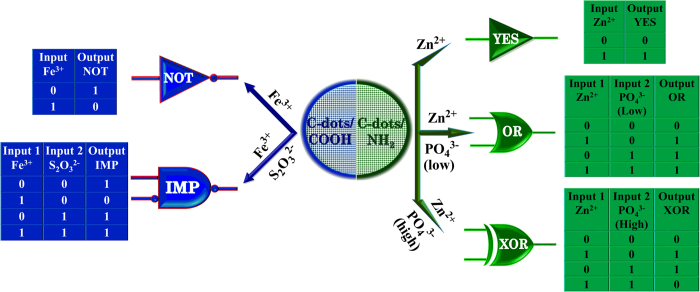
Schematic representation of logic gates obtained for C-dots1/Fe^3 + ^/S_2_O_3_^2−^ and C-dots2/Zn^2 + ^/PO_4_^3−^ systems.

Further, we demonstrated the construction of IMP logic gate by using Fe^3 + ^ (fixed concentration) and S_2_O_3_^2−^ (varying concentration) as input 1 and 2 to the C-dots1. Before obtaining the IMP logic gate with the presence of input 1 and 2, it is essential to examine the influence of individual input on C-dots and their corresponding output fluorescence intensity signal while the absence of other input. The fluorescence intensity of the C-dots1 was quenched in the presence of individual Fe^3 + ^ input and not much varied by the addition of low or high concentration of individual S_2_O_3_^2−^ input (Fig. 7). Interestingly, in the presence of both inputs, the decrease in the fluorescence intensity upon addition of Fe^3 + ^ is recovered by the addition of S_2_O_3_^2−^ (low or high concentration). These observations clearly confirm the IMP logic gate behavior^40^,^41^ according to truth table (Fig. 8).

**Figure 9:**
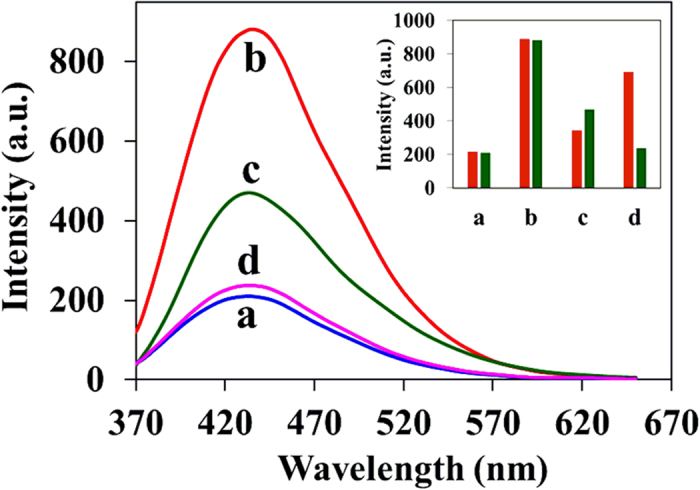
Fluorescence response of C-dots2 in the absence and presence of Zn^2 + ^(2.5 × 10^−4^ M) and PO_4_^3−^ (3.50 × 10^−3^ M); **a**: C-dots2, **b**: C-dots2/Zn^2 + ^, **c**: C-dots2/PO_4_^3−^, d: C-dots2/Zn^2 + ^/PO_4_^3−^. Inset: Bar diagram showing the fluorescence intensity changes for different combination of inputs, Red color: in the presence of low concentration of PO_4_^3−^ (1.0 × 10^−4^ M), Green color: in the presence of high concentration of PO_4_^3−^ (3.50 × 10^−3^ M).

Similarly, we demonstrated the construction of the OR and XOR logic gates with C-dots2 in the presence of Zn^2 + ^(fixed concentration) as input 1 along with PO_4_^3−^ (low and high concentration) as input 2 (Fig. 9). It was observed that, the fluorescence intensity of C-dots2 was enhanced in the presence of individual inputs Zn^2 + ^or different (low or high) concentration of PO_4_^3−^. Surprisingly, in the presence of both inputs (Zn^2 + ^and low concentration of PO_4_^3−^), the enhanced fluorescence intensity of C-dots2 due to Zn^2 + ^input was slightly decreased due to sequential addition of low concentration of PO_4_^3−^. However, the quenched fluorescence intensity was still higher compared to that of C-dots2 without any individual inputs, i.e., the output is 1 (high), these results confirm the operation of OR gate. On the contrary, the fluorescence intensity was quenched drastically upon addition of high concentration of PO_4_^3−^ indicating that the output is 0 (low) in contradiction to the function of OR gate. These results show that the use of low or high concentration of PO_4_^3−^ as input 2 along with Zn^2 + ^as input 1 generated OR or XOR logic gate functions^42^,^43^ respectively (Fig. 8), revealing that the different logic operation can be achieved by varying the concentration of inputs. We have repeatedly performed each logic gate operation for at least four times and the efficiency varied between 80–85% depending upon the chemical inputs. Based on all these results, we propose that the role of C-dots will be of great importance in the field of molecular electronic and nanodevices.

## Conclusions

We performed the detection of metal ions and anions done by carboxylate/amine functionalized C-dots sensors in pH 7.2 HEPES-buffered water solution. It was found that the functional groups present on the C-dots surface showed certain association affinity towards specific metal ions. The results revealed that both C-dots exhibit highly selective and sensitive towards the detection of metal ions such as Fe^3 + ^and Zn^2 + ^, and anions like S_2_O_3_^2−^ and PO_4_^3−^. We have also demonstrated C-dots based molecular logic gates operation using metal ions and anions as the chemical input. The multiple logic gates such as YES, NOT, IMP, OR and XOR were constructed by the consecutive processes of association of metal ion with C-dots, and dissociation by anion. This carbon-dots sensor can be utilized as various logic gates at the molecular level and it will show better applicability for the next generation of molecular logic gates. Their promising properties of C-dots may open up a new paradigm for establishment of the chemical logic gates via fluorescent chemosensors.

## Methods

### Materials

Rice was purchased from the local market of Taipei, Taiwan. Ethylenediamine and 4-(2-hydroxyethyl)-1-piperazineethanesulfonic acid were purchased from ACROS. Metal chlorides/nitrates and sodium salt of anions were purchased from Aldrich and ACROS. All commercially available organic and inorganic reagents were used without further purification. Milli-Q ultrapure water was used throughout the experiments.

### Instrumentations

UV–Visible absorption spectra were recorded on a Thermo Scientific evolution 220 UV-Visible spectrophotometer. Fluorescence spectra were recorded on a Perkin-Elmer LS45 spectrophotometer. Infrared spectra were recorded on a Thermo Scientific Nicolet iS5 FT-IR Spectrometer. TEM observations were conducted on a Hitachi H-7100 transmission electron microscope. Wide-angle X-ray powder diffraction patterns were recorded on a PANalytical (X’Pert PRO) diffractometer using Cu Kα radiation (wavelength λ = 0.1541 nm). Elemental analysis was conducted on Elementar Vario EL-III (for NCH) and EL cube (for O). All measurements were recorded at ambient temperature.

### Synthesis of C-dots

C-dots were synthesized from rice (as carbon source) by using microwave-assisted method. In a typical procedure, certain amount of rice was rinsed (rinsed repeatedly until the rinse water is clear) with Millipore water to remove the dust particles. Then that rice was added into Millipore water; stirred well for 1 h and filtered using Whatmann filter paper. The filtrated solution (rice water) was used as a precursor. The solution (10 mL) was heated in a domestic microwave oven (Panasonic, NN-ST342) at varying the power (220, 550 and 800 W) and time (5, 10 and 15 min). During microwave heating, the solution color was changed from colorless to pale brown color which results in the formation of C-dots. The obtained product was dispersed in Millipore water and then centrifuged (10000 rpm, 15 min) to remove large size particles. The supernatant solution was dialyzed against Millipore water using a dialysis membrane with MWCO of 3.5 – 5.0 kD (Spectra/Por Float-A-Lyzer G2) for two days. Finally the obtained pure C-dots were used for characterization and experimental studies. For the synthesis of amine functionalized C-dots, EDA was added into rice water and stirred well. This solution was used as a precursor and rest of synthesis procedure is same as above mentioned. Here we optimized the synthesis conditions such as heating power and reaction time to obtain the high fluorescence efficiency of C-dots. The synthesis conditions were optimized based on the fluorescence quantum yield and the higher quantum yield was obtained for the condition of 550 W and 15 min.

### General procedure for sensing of metal ion and anions

C-dots, metal ions and anions stock solutions were prepared in pH 7.2 HEPES-buffered water. The fluorescence titration measurements were carried out by adding small volumes (up to 5 μl) of the metal ion/anions to the C-dots solution in a quartz cuvette. After an addition of metal ion to the cuvette, the solution was shaken well and kept 1 min before measurement.

### Quantum yield calculation

Quinine sulphate (0.1 M H_2_SO_4_ as solvent, Φ = 0.54) was chosen as a standard to measure the quantum yield (Φ) of the C-dots. The quantum yield was determined at an excitation wavelength of 360 nm by the equation, 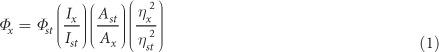 where *Φ* is the quantum yield, *I* is the fluorescence intensity, *A* is the absorbance at excitation wavelength and η is the refractive index of the solvent. The subscript *st* refers to standard with known quantum yield and *x* refers to the sample. The absorbance of standard and carbon dots solutions were kept below 0.05.

## Additional Information

**How to cite this article**: Dhenadhayalan, N. and Lin, K.-C. Chemically Induced Fluorescence Switching of Carbon-Dots and Its Multiple Logic Gate Implementation. *Sci. Rep.* **5**, 10012; doi: 10.1038/srep10012 (2015).
